# Safety and Feasibility of Transanal Endoscopic Surgery for Diffuse Cavernous Hemangioma of the Rectum

**DOI:** 10.1155/2019/1732340

**Published:** 2019-06-19

**Authors:** Ziwei Zeng, Xianrui Wu, Junji Chen, Shuangling Luo, Yujie Hou, Liang Kang

**Affiliations:** ^1^Department of Colorectal Surgery, The Sixth Affiliated Hospital, Sun Yat-sen University, Guangzhou, China; ^2^Guangdong Institute of Gastroenterology, The Sixth Affiliated Hospital, Sun Yat-sen University, Guangzhou, China; ^3^Guangdong Provincial Key Laboratory of Colorectal and Pelvic Floor Diseases, The Sixth Affiliated Hospital, Sun Yat-sen University, Guangzhou, China

## Abstract

**Purpose:**

To evaluate the safety and feasibility of transanal endoscopic surgery for diffuse cavernous hemangioma of the rectum (DCHR).

**Methods:**

All DCHR patients who underwent transanal endoscopic surgery in our hospital between January 2014 and June 2018 were reviewed.

**Results:**

A total of 7 patients with a diagnosis of DCHR underwent transanal endoscopic surgery during the study period. Four patients (57.1%) were male, with a mean age at surgery of 34.5 ± 7.7 years, and three patients (42.9%) were female, with a mean age at surgery of 29.9 ± 3.8 years. Recurrent painless rectal bleeding was the main symptom in all patients. The mean age was 32 years old (range 21-54 years). The median duration of symptoms was 10 years (range 1 month-50 years). The level of hemoglobin at admission ranged from 59.0 to 148.0 g/l (mean 106.6 g/l), and the level of mean corpuscular volume (MCV) ranged from 75.1 fl to 93.5 fl (mean 83.7 fl). Colonoscopy, computed tomography (CT), and magnetic resonance imaging (MRI) were important in the diagnosis of DCHR because of their high positive rates and accurate features. All of the lesions are between the anal canal and the descending colon. Two patients could be found with some enlarged serpentine vessels in the cervix, vagina, or corpus cavernosum by MRI. After admission, all the patients underwent transanal endoscopic surgery and four patients had simultaneous loop ileostomy. The mean operative time was 278 min (range 168-400 min). The median amount of intraoperative blood loss was 50 ml (range 10-300 ml). The mean distance from anal verge to anastomosis was 2.2 ± 0.2 cm. The anastomosis was fashioned with a stapler in two patients (28.6%). There were no intraoperative and postoperative complications. All the patients continued to recover well from the surgery, and nobody needed postoperative blood transfusions.

**Conclusions:**

The specific diagnosis rate of DCHR is low. Preoperative MRI and CT examination can make a definitive diagnosis and determine the extent of the lesions. DCHR is mostly restricted to the rectum, sigmoid colon, anal wall, and mesorectum. The best treatment for DCHR is complete lesion resection. It is safe and feasible to treat DCHR using transanal endoscopic surgery. Moreover, transanal endoscopic surgery might have a huge potential when used to treat other rectal diseases.

## 1. Introduction

Minimization of trauma has been an important direction that surgeons are pursuing without sacrificing the curability of the underlying disease. Natural orifice transluminal endoscopic surgery (NOTES) is considered the utmost form of this type of development [1]. In combination with total mesorectum excision (TME) and the single-incision laparoscopic technique, transanal total mesorectum excision (TaTME), which has been applied in rectal cancer patients, had evolved along with the concept of NOTES [2, 3]. Compared with the conventional laparoscopic procedure, TaTME is a transanal procedure which is performed from the bottom to the top and from the interior to the exterior; this confers the advantage of dealing with the rectal lesions under direct view of the disease itself [4, 5]. Moreover, it could reduce or eliminate the need for abdominal incision, which means less incision pain as well as a quicker recovery. Because of the features of TaTME, it could contribute to the precise resection of the local lesion in the mid and low rectum and in the anal canal with satisfactory outcomes [6].

Diffuse cavernous hemangioma of the rectum (DCHR) is a rare benign vascular disease. Since the first case of rectal hemangioma was reported by Phillips in 1839, there have been more than 350 cases published in the literature around the world. It most commonly affects young patients, with the most common clinical symptom being episodic painless rectal bleeding [7]. Since DCHR patients lack specific symptoms, there is often a need distinguish it from hemorrhoids, colitis, portal hypertension, and blue rubber bleb nevus syndrome (BRBNS), which have multifocal venous malformations of the skin, soft tissues, and gastrointestinal tract [7–10]. As to the management of DCHR, surgery is usually inevitable when angiographic embolization is invalid [7, 11]. Previously, we presented a case diagnosed with DCHR who underwent TaTME, but in this study we have replaced TaTME with transanal endoscopic surgery (TAES) when we used this technique to treat other rectal diseases excluding rectal cancer. This study is a natural extension of our case report with the accumulation of more cases. The aim of this study was to evaluate the safety and feasibility of TAES in the treatment of DCHR.

## 2. Materials and Methods

All DCHR patients who underwent the TAES procedure by Dr. Liang Kang between August 2016 and June 2018 were reviewed. Patients without rectal lesions were excluded. This study was approved by the institutional review board.

The clinical information, including sex, age, body mass index (BMI), symptoms, duration of symptoms, previous medical history, family history, misdiagnosis, hemoglobin (HGB) and mean corpuscular volume (MCV) on admission, and digital examination, were collected. The consequence of assistant examinations such as colonoscopy, rectal ultrasonography, computed tomography (CT), magnetic resonance imaging (MRI), and angiography (DSA) were reviewed. The process of treatment, including the length of operation, amount of intraoperative blood loss, amount of intraoperative blood transfusion, intraoperative and postoperative complications, and the postoperative recovery situation, were collected and reviewed. Follow-up investigations were conducted by face-to-face communication in the outpatient department of our hospital and by telephone communication.

### 2.1. TAES Procedure

**Figure 1:**
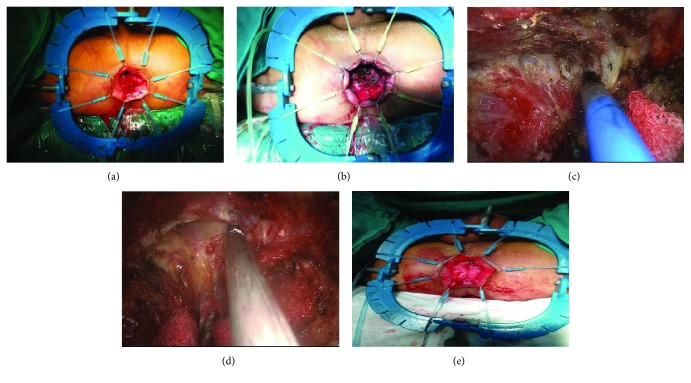
Procedure of TAES. (a) Expose the anorectum. (b) Purse strings to occlude rectal lumen. (c) Free lesions. (d) Transabdominal groups meeting with transanal groups. (e) Hand-sewn anastomosis.

The patients were placed in an extended lithotomy position. After abdominal and perineal disinfection, two groups of surgeons, the transabdominal group and the transanal group, operated simultaneously. After digital anal dilation, the Lone Star® Retractor System (CooperSurgical™ Inc., Trumbull, CT, USA) was used to sufficiently expose the anorectum (Figure 1(a)), then 2-0 Vicryl (Ethicon™, Cincinnati, OH, USA) purse strings were placed to tightly occlude the rectal lumen close to the inferior edge of the hemangioma (Figure 1(b)). But if the focus involved an anal verge, the lesions were under a circular incision directly and was kept 1 cm from the lesion to the purse strings. After isolating the lesion, the inferior intestinal tube of the purse strings was washed with 200 ml physiological saline. Washing the lavage lumen with a large volume of iodine to shed bacteria was necessary before opening the rectal wall. Before insufflating CO_2_ to create a peumo-anorectum (about 12-15 mmHg), SILS™ Port (Medtronic™, Watford, WD, UK) was introduced through the anus. Conventional laparoscopic instruments such as a high-definition laparoscope (KARL STORZ™, El Segundo, CA, USA), a harmonic scalpel® (Ethicon™, Cincinnati, OH, USA), and a grasper were introduced via the SILS™ Port. Then, a full-thickness circumferential dissection toward the perirectal plane (between Denonvilliers' fascia) was performed. The dissection was first started posteriorly and access to the presacral plane was attained (Figure 1(c)). The embryological plane was then extended either laterally or anteriorly in a sequence depending on specific situations, while the whole procedure progressed proximally. Until meeting with transabdominal group, the peritoneal reflection was not cut open, although it was reached anteriorly (Figure 1(d)).

The inferior mesenteric vessels (IMV) were skeletonized by the transabdominal group, and these were ligated and divided using Hem-O-Lok® Clips (Weck Corporation™, CO, USA). The medial and lateral attachments of the descending colon were then divided to the greatest degree to make sure an adequate length of colon could be pulled through the anus.

After delivering the specimen extracorporeally, an end-to-end straight stapled anastomosis treated with CDH29® (Ethicon™, Cincinnati, OH, USA) or a 2-0 Vicryl® (Ethicon™, Cincinnati, OH, USA) hand-sewn anastomosis (Figure 1(e)) was performed.

### 2.2. Statistical Analysis

Descriptive statistics were computed for all variables. These included means and standard deviations (SD) or medians and ranges for continuous factors and frequencies for categorical factors.

## 3. Result

Data from a total of 7 cases of DCHR who underwent TAES were collected (Table 1). Recurrent painless rectal bleeding was the main symptom in all the 7 patients. Four patients (57.1%) were male, with mean ages at diagnosis and surgery of 34.5 ± 7.7 years and 34.5 ± 7.7 years, respectively. The median duration from symptoms to diagnosis was 10 years (range 1 month-50 years). The mean levels of hemoglobin and corpuscular volume at admission were 106.7 ± 12.7 g/l and 83.8 ± 2.9 fl, respectively. Among all patients, four patients had a history of blood transfusion. The mean BMI was 19.4 ± 1.1. Family history was not reported by any of the patients.

**Figure 2:**
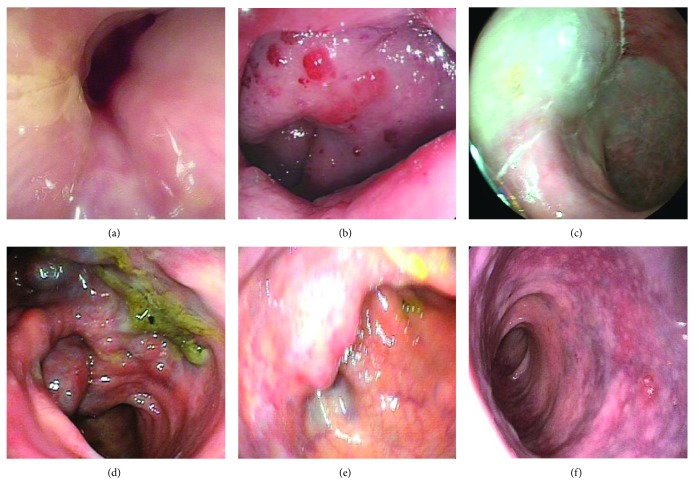
Colonoscopic findings of DCHR. (a) Normal. (b) Congestion, swelling, and bleeding. (c) Bluish swelling. (d) Enlarged serpentine vessel. (e) Submucosal nodule. (f) Bluish and purple change.

**Figure 3:**
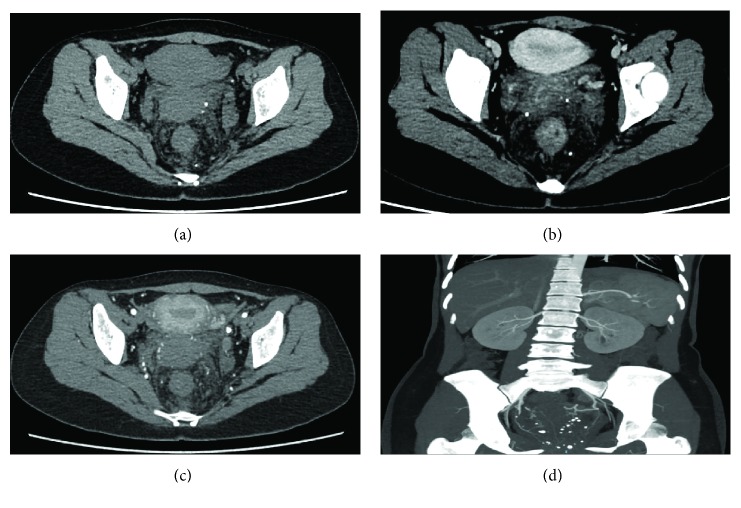
CT findings of DCHR. (a) Thickened rectal wall. (b) Enlarged serpentine vessels in the rectal wall. (c) Multiple calcification. (d) Serpentine vessels and multiple calcification.

**Figure 4:**
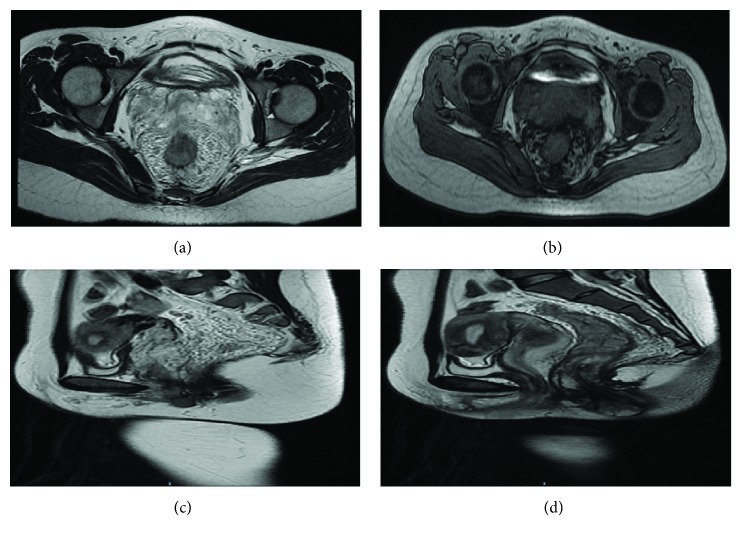
MRI findings of DCHR. (a) Thickened rectal wall and serpentine vessel. (b) Enlarged and serpentine vessel. (c) Serpentine vessel. (d) Thickened rectal and anal canal wall.

Five patients (71.4%) were once misdiagnosed with hemorrhoids and underwent hemorrhoidectomy accordingly, without improvement in their symptoms afterwards. The other two patients (28.6%) were misdiagnosed with rectal neoplasm. All patients received digital rectal examination, with bright red blood on the glove being shown in all patients (100%), rectal soft nodules being touched in 3 (42.8%), and stiff anorectal mucosa being touched in 1 (14.3%). Six patients received colonoscopy (Figure 2), which showed congestion and swelling of mucosa with a bluish or purple color. CT (Figure 3) scan was performed in 6 patients and MRI (Figure 4) in 7. The typical findings were a thickened rectum and an enlarged serpentine vessel in the rectal mesentery, and two patients were found to have some enlarged serpentine vessels in the cervix, vagina, or corpus cavernosum. The lesions of all the 7 patients originated from the dentate line to the sigmoid colon. Four patients who underwent DSA previously were found to have enlarged serpentine vessels.

All patients underwent the hybrid TAES procedure (Table 2), with a mean operation time of 278.1 ± 31.1 min. The median amount of intraoperative blood loss was 50 ml (range 10-300 ml). Two patients underwent intraoperative blood transfusion. Four patients had protective loop ileostomy. The mean distance from the anal verge to the anastomosis was 2.2 ± 0.2 cm. The anastomosis was fashioned with a stapler in two patients (28.6%). No intraoperative complication occurred. All patients continued to recover well from the surgery. Postoperative blood transfusions were not required. The median length of bowel function return was 4 days (range 2-8 days). The median length of hospital stay after surgery was 11 days (range 5-21 days). The postoperative histopathologic examination of the 7 resected specimens confirmed the diagnosis of DCHR and showed an increased amount of dilated blood vessels in the submucosa, muscular layer, serosa, and perirectal fat. These blood vessels were covered by a thin monolayer of epithelial cells and filled with red blood cells in the cavity.

None of the patients missed follow-up, with a median follow-up time of 13 months (range: 1 months to 24 months). Ileostomy closure was done successfully in 3 patients, and another patient is waiting for the procedure. Painless rectal bleeding disappeared in all the patients.

## 4. Discussion

Diffuse cavernous hemangioma of the rectum (DCHR) is a rare benign vascular lesion, which originates from the submucosal vessel and is caused by the abnormal development of the mesoderm tissue during the embryo period [12]. According to previous studies [7, 11, 13–16], the most reported chief symptom of DCHR is recurrent painless rectal bleeding. Due to the rareness of the disease entity and the lack of specific symptoms, most patients need a relatively long time to make a definitive diagnosis. In our study, the median duration of patients having a diagnosis of DCHR was 10 years.

It was reported that an abdominal X-ray is an important method for the diagnosis of diffuse cavernous hemangioma of the rectum, and in most patients closely arranged phleboliths have been observed [17]. With the development of colonoscopy, CT, rectal ultrasound, and MRI, the abdominal X-ray has been replaced by these. Colonoscopy is an important examination for affirming DCHR and observing the length of the lesions. The typical findings of colonoscopy are congestion and swelling of the blue rectal mucosa and bluish purple submucosal mass representing the enlarged tortuous vessels [18, 19]. CT is another important examination for confirming DCHR. The representative findings of CT are a thickened rectal wall, an enlarged serpentine vessel around the rectum, and sometimes multiple calcifications. In our study, five patients had these findings. In contrast to colonoscopy, it is difficult to estimate the exact extent of lesions using CT; however, CT can be used to observe the condition of other organs located in the abdomen. MRI may be an essential examination for the diagnosis of DCHR; it is superior to CT due to its capability to produce high-resolution and multidimensional images of soft tissue [20]. Therefore, it can give accurate information about the exact dimensions of DCHR, sphincteric involvement, and the involvement of adjacent structures. Some studies reported that the specific signal intensity of DCHR in MRI is hypointensity and equi-intensity in the T1W image and hyperintensity in the T2W image, and obviously progressive enhancement [8]. Moreover, the thickened rectal wall and enlarged serpentine vessel around the rectum can also be found in MRI. In this study, enlarged serpentine vessels in the cervix, vagina, or corpus cavernosum were found in two patients, but we did not find any lesions in the skin and gastrointestinal tract; thus, we did not consider it as BRBNS but only varicosity in these tissues. DSA can estimate the blood supply artery of DCHR. Obviously, leakage of contrast agents could be found when acute blood loss occurs, and contrast agents may be retained during chronic blood loss. In the vein stage, multiple tortuous veins could be found [21]. Sometimes, patients can be treated by angiographic embolization during the time of DSA. Above all, DCHR needs to be evaluated by multiple examinations to make a definitive diagnosis.

Nonoperative treatments for DCHR have been reported, and they can decrease the amount and frequency of rectal bleeding; sometimes, the rectal bleeding can disappear for a long time [15, 16, 20, 22, 23]. However, the lesions cannot be removed by nonoperative therapies, and rectal bleeding eventually recurred in most patients who received surgery. Therefore, nonoperative treatments are not recommended for curing DCHR. Different operative treatments to DCHR have been reported. Wang et al. reported 13 patients who had open surgical resection for DCHR, showing that six cases had simultaneous loop ileostomy; the median operative time was 295 min (range = 195‐400 min), the median amount of intraoperative blood loss was 793 ml (range = 50‐3000 ml), and the median amount of intraoperative blood transfusion was 1013 ml (range = 0‐3400 ml) [7]. Leal et al. showed two cases of laparoscopic-assisted bowel resections for DCHR [11]. Considering that some DCHR cases originate from the dentate line and the difficulty of the TME surgery for DCHR in resecting completely the lesions in the distal rectum, the TaTME procedure might be an alternative surgical procedure to cure DCHR. The safety and feasibility of TaTME in treating rectal cancer have been demonstrated. Zhang et al. reported on a rectal cancer patient who underwent pure-TaTME [24], and Kang et al. demonstrated the safety and feasibility of the pure-TaTME procedure in rectal cancer [5]. With the goal of minimal invasive surgery, TaTME gains access to the rectal disease through the disease itself, especially pure-TaTME which requires no abdominal incisions. When we use this technique to treat other rectal diseases except rectal cancer, transanal endoscopic surgery may be more appropriate than TaTME because TME is only used to treat mid-lower rectal cancer. In our study, all patients had undergone laparoscopic-assisted TAES, and all of them recovered satisfactorily; one was reported by Wu et al. [25]. At present, all patients had no complications after surgery. Our study shows that TAES might be an effective technique in the management of DCHR, and it is safe and feasible for the treatment DCHR.

There were some limitations of our study. Four patients had simultaneous loop ileostomy, so the length of operation may not show the true length of TAES. Our result is a retrospective analysis, and the sample size is still relatively small. Thus, the criteria of TAES to treat DCHR cannot be confirmed.

## 5. Conclusion

The specific diagnosis rate of DCHR is low. Preoperative MRI and CT examination can make a definitive diagnosis and determine the extent of the lesions. DCHR is mostly restricted to the rectum, sigmoid colon, anal wall, and mesorectum. The best treatment for DCHR is complete lesion resection. It is safe and feasible to cure DCHR using TAES. Moreover, the transanal procedure (TAES) may have a huge potential when used to treat other rectal diseases.

## Figures and Tables

**Table 1:** Patient characteristics.

Case	Sex	Age	BMI	Main symptom	Age at the onset of symptom (year)	HB on admission	MCV on admission	Misdiagnosis	Previous medical history	Digital rectal examination
1	F	25	15.05	Recurrent painless rectal bleeding	19	103	88	Hemorrhoids	Had hemorrhoidectomy	Normal
2	F	26	21.48	Recurrent painless rectal bleeding	6	67.8	75.1	Hemorrhoids	Had hemorrhoidectomy	Normal
3	M	21	16.98	Recurrent painless rectal bleeding accompany anus lump prolapse	11	59	75.7	Hemorrhoids	Had hemorrhoidectomy	Stiff anorectal mucosa
4	M	54	23.05	Recurrent painless rectal bleeding	4	139	93.5	Hemorrhoids	Had hemorrhoidectomy and upper rectal artery embolization	Soft node
5	M	23	18.68	Recurrent painless rectal bleeding	22	148	91.4	Hemorrhoids	Had hemorrhoidectomy	Normal
6	M	40	19.36	Recurrent painless rectal bleeding	10	120	76.8	Rectal cancer	No	Soft node above the dentate line
7	F	37	21.48	Recurrent painless rectal bleeding	37	110	86	Rectal neoplasm	No	Soft node

**Table 2:** Detailed treatment information of the 7 patients.

Cases	Length of operation (min)	Intraoperative blood loss (ml)	Ileostomy	Distance from anal verge to anastomosis (cm)	Anastomosis	Length of time before bowel function returned (day)	Length of hospital stay after surgery (day)	Preoperative blood transfusion (ml)
1	348	100	Yes	2.0	Stapler	3	8	—
2	168	50	No	2.5	Hand-sewn	8	21	800
3	330	50	Yes	1.0	Hand-sewn	4	5	700
4	400	300	Yes	3.0	Hand-sewn	2	17	—
5	240	50	No	2.5	Hand-sewn	6	13	—
6	226	50	Yes	2.5	Hand-sewn	3	9	—
7	235	10	No	2.0	Stapler	2	6	—

## Data Availability

The data used to support the findings of this study are available from the corresponding author upon request.
